# Cancer Cachexia among Patients with Advanced Non-Small-Cell Lung Cancer on Immunotherapy: An Observational Study with Exploratory Gut Microbiota Analysis

**DOI:** 10.3390/cancers14215405

**Published:** 2022-11-02

**Authors:** Taiki Hakozaki, Alexis Nolin-Lapalme, Masato Kogawa, Yusuke Okuma, Shohei Nakamura, Danielle Moreau-Amaru, Taichi Tamura, Yukio Hosomi, Haruko Takeyama, Corentin Richard, Masahito Hosokawa, Bertrand Routy

**Affiliations:** 1 Department of Life Science and Medical Bioscience, Waseda University, 2-2 Wakamatsu-cho, Shinjuku, Tokyo 1628480, Japan; 2 Department of Thoracic Oncology and Respiratory Medicine, Tokyo Metropolitan Cancer and Infectious Diseases Center Komagome Hospital, 3-18-22 Honkomagome, Bunkyo, Tokyo 1130021, Japan; 3 Centre de Recherche du Centre Hospitalier de l’Université de Montréal (CRCHUM), Montréal, QC H2X-0A9, Canada; 4 Research Organization for Nano and Life Innovation, Waseda University, 513 Wasedatsurumaki-cho, Shinjuku, Tokyo 1620041, Japan; 5 Department of Thoracic Oncology, National Cancer Center Hospital, 5-1-1Tsukiji, Chuo, Tokyo 1040045, Japan; 6 Department of Medical Oncology, Tokyo Metropolitan Cancer and Infectious Diseases Center Komagome Hospital, 3-18-22 Honkomagome, Bunkyo, Tokyo 1130021, Japan; 7 Service de Nutrition Clinique du Centre Hospitalier de l’Université de Montréal (CHUM), Montréal, QC H2X-0A9, Canada; 8 Computational Bio Big-Data Open Innovation Laboratory, National Institute of Advanced Industrial Science and Technology, 3-4-1 Okubo, Shinjuku, Tokyo 1698555, Japan; 9 Institute for Advanced Research of Biosystem Dynamics, Waseda Research Institute for Science and Engineering, 3-4-1 Okubo, Shinjuku, Tokyo 1698555, Japan

**Keywords:** non-small-cell lung cancer, cancer cachexia, immunotherapy, prognosis, gut microbiota, biomarker

## Abstract

**Simple Summary:**

Immunotherapy has revolutionized the therapeutic options for patients living with non-small-cell lung cancer (NSCLC). Despite the unprecedented results achieved through immunotherapy, a low body mass index, which is referred to as cachexia, and the bacterial composition of the gut microbiota are known factors associated with resistance. In this paper, we enrolled 113 Japanese patients with NSCLC and demonstrated that cachexia was associated with poor outcomes. Moreover, microbiota sequencing revealed that patients without cachexia had abundant bacteria that correlated with a beneficial outcome. Altogether, our results demonstrated an association between the gut microbiota and cachexia. This study provides a rationale to launch clinical trials on the outcome of shifting the microbiota composition of patients with cachexia that are receiving immunotherapy.

**Abstract:**

Cancer cachexia exerts a negative clinical influence on patients with advanced non-small-cell lung cancer (NSCLC) treated with immune checkpoint inhibitors (ICI). The prognostic impact of body weight change during ICI treatment remains unknown. The gut microbiota (GM) is a key contributor to the response to ICI therapy in cancer patients. However, the association between cancer cachexia and GM and their association with the response to ICIs remains unexplored. This study examined the association of cancer cachexia with GM composition and assessed the impact of GM on clinical outcomes in patients with NSCLC treated with ICIs. In this observational, prospective study, which included 113 Japanese patients with advanced NSCLC treated with ICIs, the prevalence of cachexia was 50.4% (57/113). The median progression-free survival (PFS) and overall survival (OS) were significantly shorter in the cachexia group than in the non-cachexia group (4.3 vs. 11.6 months (*p* = 0.003) and 12.0 months vs. not reached (*p* = 0.02), respectively). A multivariable analysis revealed that baseline cachexia was independently associated with a shorter PFS. Moreover, a gain in body weight from the baseline (reversible cachexia) was associated with a significantly longer PFS and OS compared to irreversible cachexia. Microbiome profiling with 16S rRNA analysis revealed that the cachexia group presented an overrepresentation of the commensal bacteria, *Escherichia-Shigella* and *Hungatella*, while the non-cachexia group had a preponderance of *Anaerostipes, Blautia*, and *Eubacterium ventriosum*. *Anaerostipes* and *E. ventriosum* were associated with longer PFS and OS. Moreover, a cachexia status correlated with the systemic inflammatory marker-derived-neutrophil-to-lymphocytes ratio (dNLR) and Lung Immune Prognostic Index (LIPI) indexes. Our study demonstrates that cachexia and longitudinal bodyweight change have a prognostic impact on patients with advanced NSCLC treated with ICI therapy. Moreover, our study demonstrates that bacteria associated with ICI resistance are also linked to cachexia. Targeted microbiota interventions may represent a new type of treatment to overcome cachexia in patients with NSCLC.

## 1. Introduction

Immune checkpoint inhibitors (ICIs), either as a monotherapy or in combination with platinum-doublet chemotherapy (chemo), are the standard of care for patients with advanced non-small-cell lung cancer (NSCLC) [1]. Through the rapid evolution in the management of patients with NSCLC, there has been an increased interest in the development of biomarkers to predict the response to ICIs [2]. Several reports have shown that the gut microbiota (GM) composition is a key contributor to the response to ICI therapy [3,4,5,6]. Similar to murine experiments, meta-analyses have demonstrated that altering the GM with antibiotics is associated with deleterious effects [6,7]. Moreover, GM profiling revealed that a high baseline bacterial diversity and an overrepresentation of immunogenic bacteria such as *Akkermansia muciniphila*, *Ruminococcus*, and *Anaerostipes* were associated with the response to ICI therapy in large cohorts of patients with NSCLC and melanoma [6,7,8,9]. Moreover, several strategies to shift the GM composition to increase the response to ICIs are currently being evaluated in the clinical setting [10,11], and fecal microbiota transplantation has been shown to overcome secondary resistance to ICIs.

Cancer cachexia is a multifactorial syndrome that leads to substantial weight loss, primarily due to skeletal muscle loss. Studies suggest that the pathophysiology of cancer cachexia involves crosstalk between skeletal muscle and immune cells across multiple organs, such as adipose tissue, the brain, liver, heart, and the gastrointestinal tract [12,13]. Inflammatory cytokines, such as tumor necrosis factor-alpha, interleukin-1, interleukin-6, and interferon-gamma, are considered key mediators in the development of cancer cachexia [14]. These cytokines, which potentiate a systemic inflammatory response, are associated with reduced food intake and a suppressed appetite [15]. Accumulating evidence supports the role of the GM in appetite regulation, nutrient metabolism, and various diseases [16,17,18,19]; however, the relationship between cancer cachexia and the GM among patients with cancer has not been fully explored.

Cancer cachexia can have a substantially negative impact on the response to ICI therapy and a patient’s quality of life [20,21,22,23]; importantly, most of these studies have focused on the patient’s initial weight, and the change in weight over time in patients treated with ICIs was not considered.

The objectives of our study were to examine whether cancer cachexia is associated with GM composition and to assess the impact of the GM on the clinical outcomes in patients with advanced NSCLC treated with ICI therapy.

## 2. Materials and Methods

### 2.1. Patients and Clinical Outcomes

We conducted a prospective observational study at a single institution. Patients who fulfilled all of the following criteria were eligible for this study: (1) histologically or cytologically confirmed to have unresectable advanced (stage III or IV) or recurrent (after definitive treatment) NSCLC and (2) a history of ICI (nivolumab, pembrolizumab, and atezolizumab) alone or in combination with platinum-doublet chemo (ICI + chemo) at recommended doses either as the first-line or later-line therapy at Tokyo Metropolitan Cancer and Infectious Diseases Center, Komagome Hospital (Tokyo, Japan). All patients provided written informed consent before their entry into the trial. The study was registered with the UMIN Clinical Trials Registry (ID: UMIN000021734).

By conducting a post hoc analysis, we additionally analyzed the relationship between the presence of cancer cachexia and prognosis in those who received ICI therapy, as well as the characteristics of the GM in patients with cancer cachexia.

In accordance with the international consensus presented by the European Palliative Care Research Collaborative in 2011 [24], comorbidity with cancer cachexia was defined as the following: (1) involuntary weight loss >5% over the past 6 months or (2) body mass index (BMI) <20 kg/m^2^ and involuntary weight loss >2% over the past 6 months. In addition, according to the change in the patients’ longitudinal body weight during ICI therapy, we divided patients with cachexia at baseline into “cachexia-reversible” and “cachexia-irreversible” groups. The “cachexia-reversible” group constituted those who experienced >5% (>2% for BMI <20 kg/m^2^) weight gain compared with baseline during ICI therapy; we categorized the rest into the “cachexia-irreversible” group. Similarly, we divided patients without cachexia at baseline into “cachexia-latent” and “cachexia-free” groups. The “cachexia-latent” group comprised those who experienced >5% (>2% for BMI <20 kg/m^2^) weight loss during ICI therapy; we categorized the rest as the “cachexia-free” group.

We defined progression-free survival (PFS) as the time from the start of ICI therapy to the first documented instance of disease progression (PD) or the date of death. Overall survival (OS) was determined from the date of starting ICI therapy to the date of death, irrespective of the cause of death. Responses to the treatment were evaluated by the investigators using computed tomography images according to the revised Response Evaluation Criteria in Solid Tumors guideline version 1.1 (RECIST version 1.1) [25]. Patients without PD or who died at the time of the analysis were censored at the date of the last contact. Continuation of ICI therapy beyond RECIST PD was allowed. Adverse drug reactions induced by ICI treatment were monitored according to the National Cancer Institute Common Terminology Criteria for Adverse Events (NCI-CTCAE) version 5.0 until the first documented instance of PD or the date of death. Antibiotic use (irrespective of the spectrum, administration routes, or duration) within one month before the start of ICI therapy was prospectively recorded for all patients. Moreover, baseline dNLR and LIPI were calculated as previously published [26,27].

### 2.2. Fecal Samples and DNA Extraction

Fecal samples at the baseline (within one week before or after starting ICI therapy) were prospectively collected using a commercial sampling kit containing guanidine solution (TechnoSuruga Laboratory Co., Ltd., Shizuoka, Japan). The stool samples were immediately stored at 4 °C and frozen at −80 °C within 24 h. At the time when certain samples were collected, genomic DNA was extracted from the stool samples using the NucleoSpin^®^ DNA stool kit (Catalog number: 740,472.10, Macherey-Nagel GmbH and Co. KG, Düren, Germany) and immediately stored at −80 °C.

### 2.3. 16S rRNA Gene Next-Generation Sequencing Analysis

Isolated DNA was analyzed using 16S ribosomal RNA (rRNA) gene (16S rDNA) sequencing to investigate the microbial community in fecal samples. The V3–V4 hypervariable regions of the 16S rDNA were amplified using a prokaryotic universal polymerase chain reaction (PCR) primer pair (forward: 5′-ACACTCTTTCCCTACACGACGCTCTTCCGATCT-3′, reverse: 5′-GTGACTGGAGTTCAGACGTGTGCTCTTCCGATCT-3′) (TechnoSuruga Laboratory Co., Ltd.). In addition to the V3–V4 specific priming regions, these primers were complementary to standard Illumina forward and reverse primers. To reduce the formation of spurious byproducts during the amplification process, the touchdown PCR method for thermal cycling was used with a Rotor-Gene Q quantitative thermal cycler (Qiagen, Hilden, Germany). Sequencing was conducted using a paired end, 2 × 250-bp cycle run on an Illumina MiSeq sequencing system and MiSeq Reagent Nano Kit version 2 (500 cycles) chemistry.

### 2.4. Bioinformatics Analysis

The obtained sequence reads were processed using Quantitative Insights into the Microbial Ecology 2 (QIIME2) (version 2021.11) pipeline [28]. The DADA2 R package (version 1.8.0) [29] was used to generate exact amplicon sequence variants (ASV) of each sample from raw amplicon sequences. The taxonomy assignment was performed against the SILVA reference database (version 138) and genus-level assignments were based on exact matching between ASV and reference sequences. To quantitatively measure alpha diversities, Faith’s phylogenetic diversity (FaithPD) index and the Shannon index were calculated for each sample. The significance of differences among the different groups was evaluated using the Mann–Whitney U test. To quantitatively measure beta diversity, Bray–Curtis distance values were calculated. Permutational multivariate analysis of variance (PERMANOVA) was used to assess the significance of sample groupings using 999 Monte Carlo simulations. Finally, linear discriminant analysis effect size (LEfSe) [30] was determined to identify bacterial features differentially represented in patients with and without cancer cachexia at the genus level.

### 2.5. Statistical Analysis

We used descriptive statistics to summarize the patients’ baseline characteristics. Between-group differences were assessed using Fisher’s exact tests for categorical data and the Mann–Whitney U test for continuous variables. We estimated the survival distributions (PFS and OS) using the Kaplan–Meier method and compared the differences between the groups using a log-rank test. The predictors of survival were explored using Cox regression. Characteristics with a *p*-value of <0.05 after the univariate analysis were included in the multivariate analysis. The overall response rate was defined as the proportion of patients with complete or partial response as their best overall response according to RECIST 1.1. All *p*-values in this study were two-sided, and *p* < 0.05 was considered statistically significant. All statistical analyses were performed with R (version 4.1.2, R Foundation for Statistical Computing, Vienna, Austria).

## 3. Results

### 3.1. Patient Characteristics

We enrolled 113 patients with advanced NSCLC who received ICI alone or in combination with platinum-doublet chemotherapy. The median age was 70 (range: 31–86) years and the median follow-up was 10.8 months. Regarding ICI therapy, 73 (64.6%) and 40 (35.4%) patients were treated with ICI monotherapy and ICI + chemo, respectively. The combination of cisplatin, pemetrexed, and pembrolizumab was the most frequent platinum-doublet used in the chemo-IO group. Seventy-nine (69.9%) and thirty-four (30.1%) patients received ICI as first-line and second- or later-line treatment, respectively. Twenty-two (20.2%) patients received antibiotics within one month before starting ICI therapy. Among the 113 patients, 57 (50.4%) had cachexia prior to ICI initiation (Figure S1). The baseline characteristics were well-balanced between patients with and without cachexia except for the proportion of patients treated with ICI + chemo, which was higher in the non-cachexia group (Table 1). When we analyzed the patients according to the timing of the ICI therapy, cancer cachexia was more prevalent in those who received second- or later-line treatment (64.7% (22/34)) than in treatment naïve patients (44.3% (35/79)).

### 3.2. Outcomes of ICI Therapy in the Subgroups with and without Cachexia at Baseline

**Figure 1:**
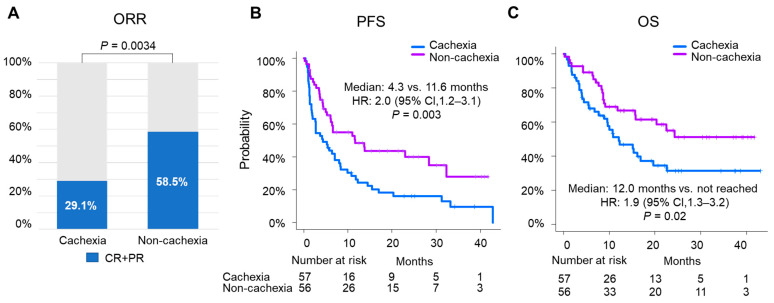

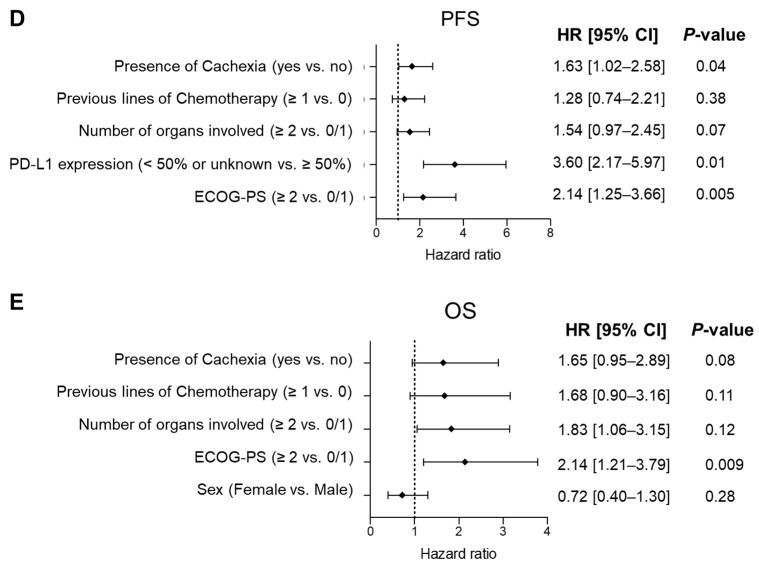
Impact of cachexia on the efficacy of ICI therapy. (**A**) Best objective response and estimated Kaplan–Meier curves for (**B**) PFS and (**C**) OS comparing patients with cancer cachexia (*n* = 57) against those without cachexia (non-cachexia) (*n* = 56) in the overall cohort. Multivariable analysis for (**D**) PFS and (**E**) OS.

In this cohort, the objective response rates for the patients with and without cachexia were 29.1% and 58.5%, respectively (*p* = 0.0034) (Figure 1A). The median PFS was 4.3 months (95% confidence interval (CI), 2.0–8.1) and 11.6 months (95% CI, 6.1–32.3) in the cachexia and non-cachexia groups, respectively (*p* = 0.003; hazard ratio (HR) 2.0; 95% CI, 1.2–3.1) (Figure 1B). The median OS was shorter in the cachexia group than in the non-cachexia group (12.0 months (95% CI, 7.7–20.0) vs. not reached (NR) (95% CI, 15.6–NR); *p* = 0.02; HR, 1.9 (95% CI, 1.1–3.2)) (Figure 1C). Similarly, for the patients treated with ICI monotherapy, the median PFS and OS were both significantly decreased in the cachexia group (*p* = 0.024 and *p* = 0.011, respectively) (Figure S2A,B).

Given the association between cachexia and outcomes, we performed a multivariate analysis accounting for standard prognostic factors. The multivariable analysis for the PFS revealed that cachexia retained its significant association (*p* = 0.04); other factors such as the number of organs involved, programmed death-ligand 1 (PD-L1) expression, and the Eastern Cooperative Oncology Group Performance Status (ECOG) also retained their significant associations (Figure 1D). The multivariate analysis for OS also showed a numerical difference for cachexia (*p* = 0.08) (Figure 1E). Altogether, these results indicate that cachexia was associated with detrimental outcomes.

### 3.3. Outcomes According to the State of Transition of Cachexia during ICI Therapy

**Figure 2:**
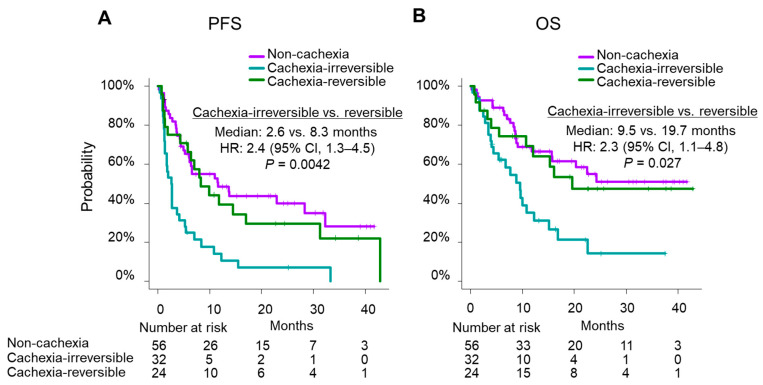
Estimated Kaplan–Meier curves for PFS and OS considering changes in the patients’ longitudinal body weight during ICI therapy; (**A**) PFS and (**B**) OS comparing the “cachexia-irreversible” and “cachexia-reversible” groups against those without cachexia at baseline.

We further explored the prognostic impact of cachexia according to the change in the patients’ longitudinal body weight during the course of ICI therapy (Figure S1). In this subgroup analysis, we observed that patients with baseline cachexia had a favorable outcome upon weight gain after ICI initiation. The median PFS of the cachexia-irreversible (*n* = 32) and cachexia-reversible (*n* = 24) groups was 2.6 months (95% CI, 1.4–4.1) and 8.3 months (95% CI, 4.3–17.0), respectively (*p* = 0.0042) (Figure 2A). The median OS was 9.5 months (95% CI, 4.1–12.3) and 19.7 months (95% CI, 10.7–NR), respectively (*p* = 0.027) (Figure 2B).

The median PFS of the cachexia-free (*n* = 16) and cachexia-latent (*n* = 39) groups was 13.7 months (95% CI, 6.4–NR) and 4.6 months (95% CI, 3.4–NR), respectively (*p* = 0.067) (Figure S3A). The median OS was NR (95% CI, 20.3–NR) and 11.7 months (95% CI, 6.3–NR), respectively (*p* = 0.059) (Figure S3B).

### 3.4. Differences in GM between the Patients with and without Cachexia at Baseline

**Figure 3:**
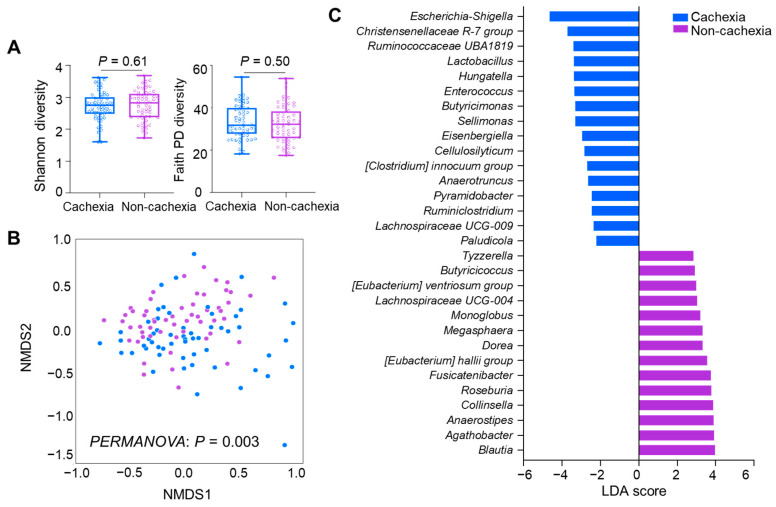
Differential analysis of GM stratified according to presence of cancer cachexia at the baseline: (**A**) alpha diversity analysis with the Shannon and FaithPD indices, (**B**) ordination method-based nonmetric multidimensional scaling plots with Bray–Curtis distance, and (**C**) differential abundance analysis with LefSe.

We then sought to determine the microbiota composition of the patients with or without cancer cachexia. First, the alpha diversity analysis revealed no significant difference in the Shannon (*p* = 0.61) and FaithPD (*p* = 0.50) indices between the two groups (Figure 3A). A beta diversity analysis using ordination method-based nonmetric multidimensional scaling plots revealed a distinct clustering of the subgroups with and without cachexia in the Bray–Curtis (*p* = 0.003) distance, which was confirmed by PERMANOVA (Figure 3B). Second, at the taxa level, the LEfSe analysis identified that the bacterial features to had different compositions in the groups with and without cancer cachexia. In patients with cachexia, *Escherichia-Shigella*, *Christensenellaceae R-7*, *Cellulosilyticum*, and *Hungatella* were preponderant (Figure 3C). Conversely, in patients without cachexia, *Anaerostipes*, *Agathobacter, Blautia, Dorea Eubacterium halli*, and *Eubacterium ventriosum* were dominant. Altogether, these results reveal that patients with or without cachexia had different GM compositions. We analyzed the compositional difference in GM between the subgroups further stratified by the change in the longitudinal body weight during ICI therapy. However, we did not observe any differences with respect to both alpha and beta diversities (Figure S4A,B). Therefore, we did not further investigate the difference in the microbiota composition in the absence of a difference in diversity indexes.

**Figure 4:**
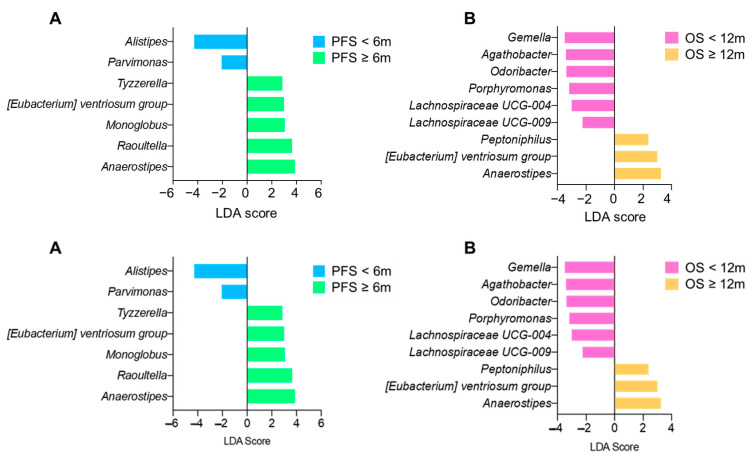
Differential abundance analysis with LEfSe between the subgroups according to PFS (**A**) and OS (**B**).

Next, we determined the differential abundance of the bacteria based on the PFS and OS regardless of their cachexia status. We observed that *Anaerostipes* and *E. ventriosum* were both increased in patients with a PFS ≥ 6 months and an OS ≥ 12 months (Figure 4A,B).

### 3.5. Associations between Inflammatory Indexes and Cachexia at Baseline

Lastly, following the observation made between cachexia and microbiota composition, we determined if a surrogate marker of inflammation could be the link between these two factors. Therefore, we compared the baseline-derived neutrophil–lypmphocyte ratio (dNLR) and lung immune prognostic index (LIPI) indexes between the patients with and without cancer cachexia. Compared with the patients without cancer cachexia, those with cachexia had significantly higher baseline dNLR (*p* = 0.004) and LIPI indexes (*p* = 0.024), both of which were associated with worse clinical outcomes (Table 2). When we compared the cachexia-irreversible and cachexia-reversible groups, no significant differences were observed in the baseline dNLR (*p* = 0.49) and LIPI indexes (*p* = 0.513) (Table 3).

## 4. Discussion

Our study demonstrates the deleterious impact of cancer cachexia in patients with advanced NSCLC amenable to ICI therapy. Our multivariable analysis revealed that the presence of cachexia at baseline was independently associated with poor PFS and OS. Moreover, we identified a subgroup of patients with baseline cachexia who were able to gain weight and reverse their cachexia status, and this was associated with favorable outcomes. Furthermore, GM profiling revealed unique bacterial clusters in the patients with and without cachexia. At the genus level, the patients without cachexia were enriched with beneficial bacteria including *Eubacterium*, *Anaerostipes*, and *Blautia*. This is one of the first studies to investigate the clinical significance of cancer cachexia in patients with advanced NSCLC treated with ICI therapy, in conjunction with a biomarker analysis focused on the GM signature of cachexia and the response to ICI therapy.

Retrospective studies have shown the negative impact of cancer cachexia on the survival of patients with advanced NSCLC treated with ICI therapy, either as PD-1/PD-L1 inhibitor monotherapy [20,21] or PD-1/PD-L1 inhibitor plus platinum-doublet chemotherapy [22,23]. These reproducible results confirmed the negative impact of cachexia in patients treated with ICIs.

Our study indicates that the change in the longitudinal body weight during ICI therapy could have a substantial prognostic impact in patients with or without cancer cachexia at baseline. Only one preceding study noted that the duration of response (DOR) with pembrolizumab monotherapy among patients who achieved complete or partial response and recovered from cancer cachexia (*n* = 10) was longer than that in patients who achieved the same but did not recover from cachexia (*n* = 3) (median DOR, NR vs. 7.4 months; *p* = 0.02) [20]. This result implies that a transition in the state of cachexia may occur during treatment and can influence the effectiveness of ICI therapy. Our results suggest that for a subgroup of patients, cachexia is reversible, further underlining the importance of a multidisciplinary team and a key role of the cancer nutritionist regarding the regular assessment of a patient’s intake and diet and the provision of guidance concerning reverse cachexia. Currently, several pharmacological interventions for cancer cachexia are being evaluated. As an additional option, a novel selective ghrelin receptor agonist, anamorelin (ONO-7643), has been approved for patients with cancer cachexia complicated with advanced NSCLC [31] or gastrointestinal cancer [32].

Recent insights from multiple cohort studies highlight the potential utility of the GM as a biomarker of the response to ICI therapy in various cancers, concerning beneficial bacteria including *A. muciniphila*, *Anaerostipes*, *Eubacterium*, and *Ruminococcus* [3,4,5,7]. Several preclinical studies identified the gut microbiome composition or specific bacterial characteristics of cancer cachexia in tumor-bearing mice models [33,34,35]. Studies also suggest that the nutritional strategy or prebiotic supplementation to modulate the GM may aid in the recovery from cachexia. [33,35]. Recently, two clinical studies detected the compositional differences in GM between two small cohorts of NSCLC patients with and without cancer cachexia [36,37]. Consistent with these studies, in our large cohort of NSCLC, we did not observe any differences in alpha diversity. However, we found a different microbial community between the patients with cachexic and non-cachexic cancer. Similarly, in a cohort of patients in the Netherlands, bacteria from the Enterobacteriaceae family had a high representation in the cachexia group. Importantly, overrepresented bacteria from the non-cachexia group, such as *Anaerostipes* and *E. ventriosum*, were abundant in patients with longer PFSs and Oss. However, due to the small number of patients in the subgroup analysis of the cachexia-reversible and cachexia-irreversible subgroups, we were not able to demonstrate a prognostic value in separating these two groups at the baseline.

Next, to strengthen the potential link between cachexia and microbiota, we demonstrated that cachexia was associated with relatively high dNLR and LIPI indexes. Importantly, these scores had been known to be correlated with worse outcomes in multiple cohorts [26,27], but these were also recently associated with microbiota composition [9]. According to a study by McCulloch et al. [9], patients in the high NLR group had abundant Gram-negative bacteria, such as *Intestinimonas massiliensis* and *Oscillibacter valericigenes* [9]. This was the first study that explored the association between cancer cachexia and systemic inflammatory indexes in combination with a GM analysis. However, similar to the microbiota analysis, there were no differences in the baseline inflammatory indexes between the cachexia-reversible and cachexia-irreversible subgroups. Therefore, a longitudinal assessment of the GM compositions in combination with systemic inflammatory markers may be required in future.

Our study had several limitations. First, this study was performed as a post hoc analysis of a single-center, prospective, observational study without a healthy control group. The study was limited to Japanese patients with advanced NSCLC; this may impair the generalizability of our findings with respect to bacterial taxa. Second, the cancer cachexia diagnosis was not performed with an evaluation of the loss of skeletal muscle using an artificial intelligence algorithm. We acknowledge the continuous evolution of the diagnostic criteria for subgroups of disease-related malnutrition including that of cancer cachexia. For this study, we used the widely accepted and quoted definition by Fearon et al. Importantly, our demonstration that the inflammatory markers—the dNLR and LIPI indexes—correlated with our definition of cachexia in part validate our findings. Third, we did not consider the patients’ dietary intake at baseline or its change during ICI therapy, which can be a substantial contributor to the GM composition or longitudinal body weight. In future analyses, sequential fecal sampling alongside the surveillance of dietary intake will provide further insight into the relationship between cachexia, GM composition, and the response to ICIs over the duration of therapy. Finally, the 16S rDNA sequencing may have been underpowered with regard to the elaborate illustration of the signature of the entire GM.

## 5. Conclusions

Our study confirms the deleterious impact of cancer cachexia on NSCLC outcomes and demonstrates that a longitudinal change in bodyweight during ICI therapy can have a substantial prognostic impact on patients with cachexia at baseline. Furthermore, we demonstrate that cachexia is associated with a unique microbiome composition. Altogether, our study elucidates key notions that advance our knowledge and support future studies on the importance of diet and microbiota interventions in patients amenable to ICI therapy.

## Figures and Tables

**Table 1:** Baseline characteristics of the enrolled patients (*n* = 113).

Characteristics	Patients without Cachexia (Non-Cachexia) (*n* = 56)	Patients with Cachexia (*n* = 57)	*p*-Value
Age			
Median (range), years	70 (31–86)	71 (47–86)	0.13
Sex, *n* (%)			
Female	18 (32.1)	23 (40.4)	0.44
Smoking status			
Brinkman index ≥ 400	42 (75.0)	38 (66.7)	0.41
Previous antibiotic use within 1 month, *n* (%)			
Yes	10 (17.9)	12 (21.1)	0.81
ECOG-PS, *n* (%)			
0/1	46 (82.1)	41 (71.9)	0.43
2	8 (14.3)	12 (21.1)	
3	2 (3.6)	4 (7.0)	
Histological subtypes, *n* (%)			
Adenocarcinoma	38 (67.9)	34 (59.6)	0.27
Squamous cell carcinoma	12 (21.4)	12 (21.1)	
NSCLC, NOS	6 (10.7)	11 (19.3)	
Staging, *n* (%)			
III	3 (5.4)	2 (3.5)	0.93
IVA	14 (25.0)	13 (22.8)	
IVB	23 (41.1)	23 (40.4)	
Recurrence	16 (28.6)	19 (33.3)	
PD-L1 expression, *n* (%)			
Negative/Unknown	18 (32.1)	16 (28.1)	0.92
1%–49%	16 (28.6)	20 (35.1)	
≥50%	22 (39.3)	21 (36.8)	
EGFR mutation status, *n* (%)			
Negative/Unknown	55 (98.2)	55 (96.5)	1.00
Positive	1 (1.8)	2 (3.5)	
Treatment regimen, *n* (%)			
ICI monotherapy	31 (55.4)	42 (73.7)	0.05
ICI + Platinum doublet	25 (44.6)	15 (26.3)	
CDDP + PEM + Pembrolizumab	12 (21.4)	8 (14.0)	
CBDCA + PEM + Pembrolizumab	5 (8.9)	4 (7.0)	
CBDCA + nab-PTX + Pembrolizumab	7 (12.5)	2 (3.5)	
CBDCA + PTX + BEV + Atezolizumab	1 (1.8)	1 (1.8)	
Prior chemotherapy, *n* (%)			
Yes	12 (21.4)	22 (38.6)	0.065
Body weight			
Median (range), kg	59.7 (37.2–95.7)	50.4 (31.7–80.3)	<0.001
Body mass index			
Median (range), kg/m^2^	22.6 (14.7–31.6)	20.5 (14.1–27.4)	<0.001
Body weight loss			
Median (IQR), kg	0.8 (0.2–2.1)	7.4 (6.2–9.5)	<0.001
Albumin			
Median (IQR), g/dL	3.9 (3.4–4.1)	3.3 (2.9–3.8)	<0.001

Abbreviations: ECOG-PS, Eastern Cooperative Oncology Group-performance status; EGFR, epidermal growth factor receptor; ICI, immune checkpoint inhibitor; IQR, interquartile range; NOS, not otherwise specified; NSCLC, non-small-cell lung cancer; PD-L1, programmed death-ligand 1; CDDP, cisplatin; PEM, pemetrexed; CBDCA, carboplatin; nab-PTX, nanoparticle albumin-bound paclitaxel; PTX, paclitaxel; BEV, bevacizumab.

**Table 2:** Comparison of the baseline inflammatory indexes between the cachexia and non-cachexia groups.

Indexs	Group	Non-Cachexia	Cachexia	*p*-Value
		(*n* = 56)	(*n* = 57)	
dNLR	dNLR < 3	43 (76.8)	29 (50.9)	0.006 *
	dNLR ≥ 3	13 (23.2)	28 (49.1)	
LIPI	0: good	32 (57.1)	22 (38.6)	0.087
	1: intermediate	19 (33.9)	23 (40.4)	
	2: poor	5 (8.9)	12 (21.1)	

Abbreviations: dNLR, derived neutrophil to lymphocyte ratio; LIPI, Lung Immune Prognostic Index. * *p* < 0.05

**Table 3:** Baseline inflammatory indexes comparing the cachexia-irreversible and cachexia-reversible groups.

Indexes	Group	Cachexia-Irreversible	Cachexia-Reversible	*p*-Value
		(*n* = 32)	(*n* = 24)	
dNLR	dNLR <3	19 (59.4)	9 (37.5)	0.176
	dNLR ≥ 3	13 (40.6)	15 (62.5)	
LIPI	0: good	15 (46.9)	6 (25.0)	0.082
	1: intermediate	9 (28.1)	14 (58.3)	
	2: poor	8 (25.0)	4 (16.7)	

Abbreviations: dNLR, derived neutrophil to lymphocyte ratio. LIPI, Lung Immune Prognostic Index.

## Data Availability

The datasets used and analyzed during the present study are available from the corresponding author on reasonable request.

## Supplementary Materials

The following supporting information can be downloaded at: https://www.mdpi.com/article/10.3390/cancers14215405/s1, Figure S1: Study flow; Figure S2: Estimated Kaplan–Meier curves for (A) PFS and (B) OS comparing patients with cancer cachexia against those without cachexia (non-cachexia) among those treated with ICI monotherapy; Figure S3: Estimated Kaplan–Meier curves for PFS and OS considering changes in the patients’ longitudinal body weight during ICI therapy; (A) PFS and (B) OS comparing the “cachexia-free” and “cachexia-latent” groups against those with cachexia at baseline.; Figure S4: Differential analysis of GM stratified according to changes in the patients’ longitudinal body weight during ICI therapy: (A) alpha diversity analysis with the Shannon and FaithPD indices, (B) ordination method-based nonmetric multidimensional scaling plots with Bray–Curtis distance.
