# Supplementary material for: Cancer Cachexia among Patients with Advanced Non-Small-Cell Lung Cancer on Immunotherapy: An Observational Study with Exploratory Gut Microbiota Analysis
Source: Cancers (Basel). 2022 Nov 2;14(21):5405. doi: 10.3390/cancers14215405 (PMC9658074; doi:10.3390/cancers14215405)
Supplement: Supplementary file 1 [file cancers-14-05405-s001.zip › cancers-1916087-supplementary.pdf]

cancers

Supplementary Materials: Cancer Cachexia among Patients with Advanced Non-Small-Cell Lung Cancer on Immunotherapy: An Observational Study with Exploratory Gut Microbiota Analysis

Taiki Hakozaki, Alexis Nolin-Lapalme, Masato Kogawa, Yusuke Okuma, Shohei Nakamura, Danielle Moreau-Amaru, Taichi Tamura, Yukio Hosomi, Haruko Takeyama, Corentin Richard, Masahito Hosokawa and Bertrand Routy

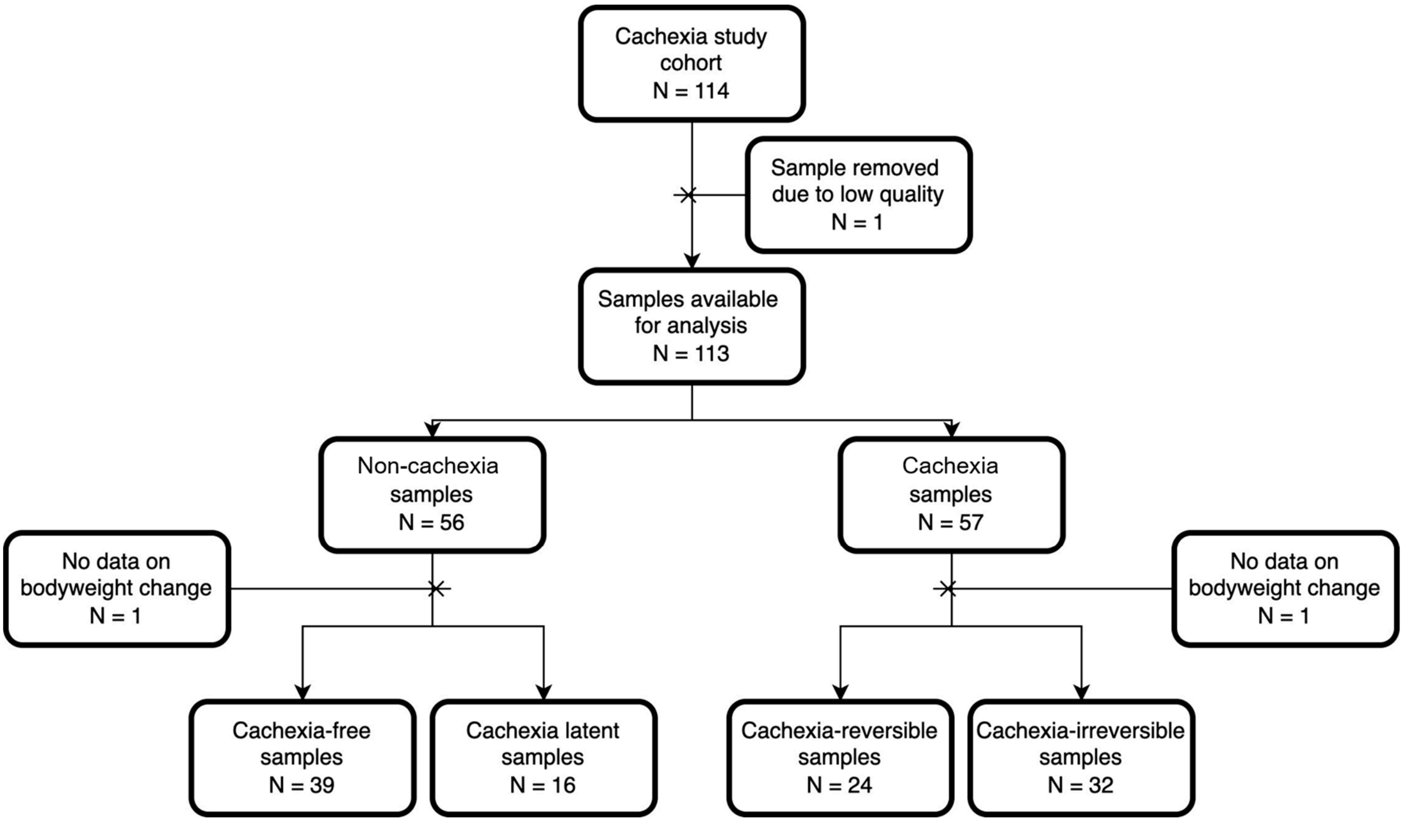

Figure S1. Study flow.

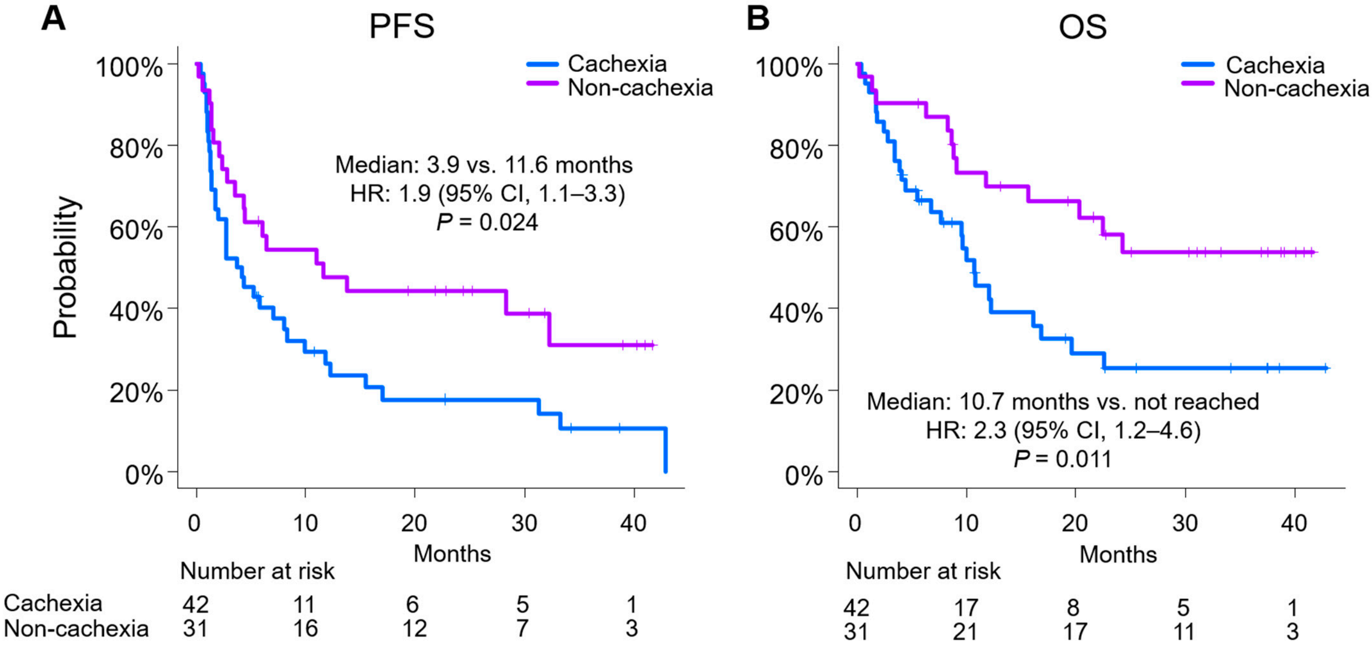

Figure S2. Estimated Kaplan–Meier curves for (**A**) PFS and (**B**) OS comparing patients with cancer cachexia against those without cachexia (non-cachexia) among those treated with ICI monotherapy.

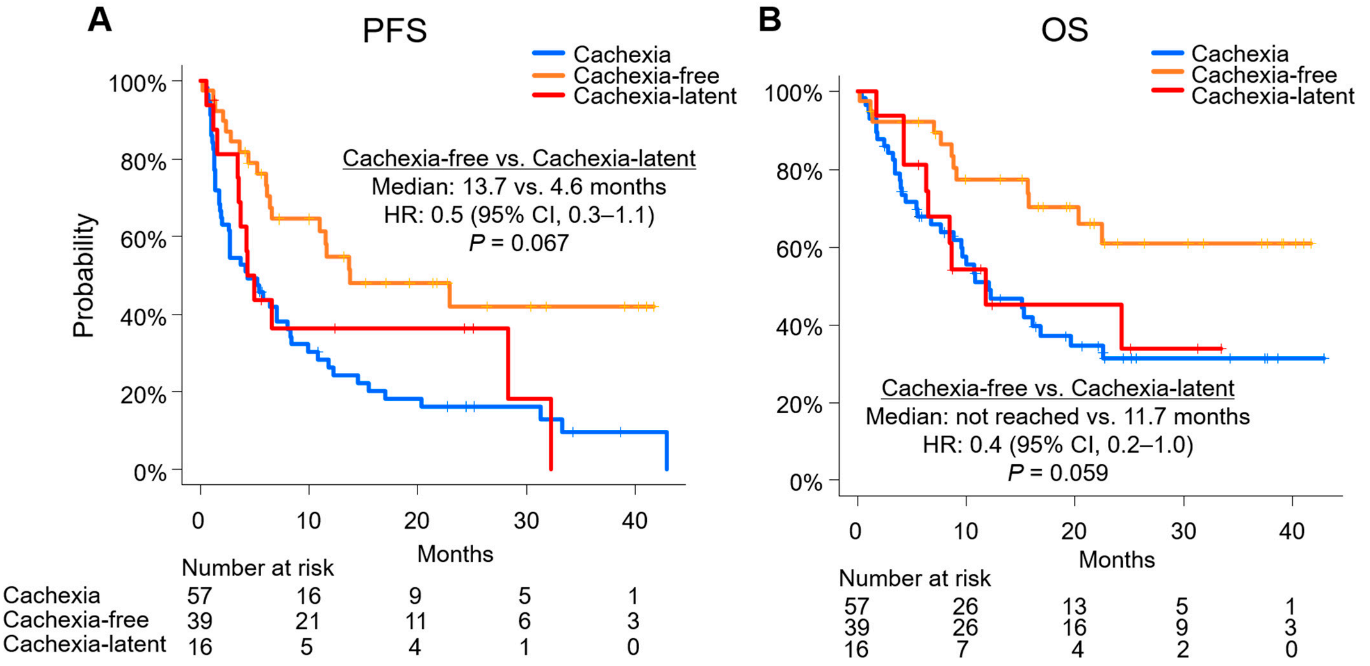

Figure S3. Estimated Kaplan–Meier curves for PFS and OS considering changes in the patients' longitudinal body weight during ICI therapy; (**A**) PFS and (**B**) OS com-paring the "cachexia-free" and "cachexia-latent" groups against those with cachexia at baseline.

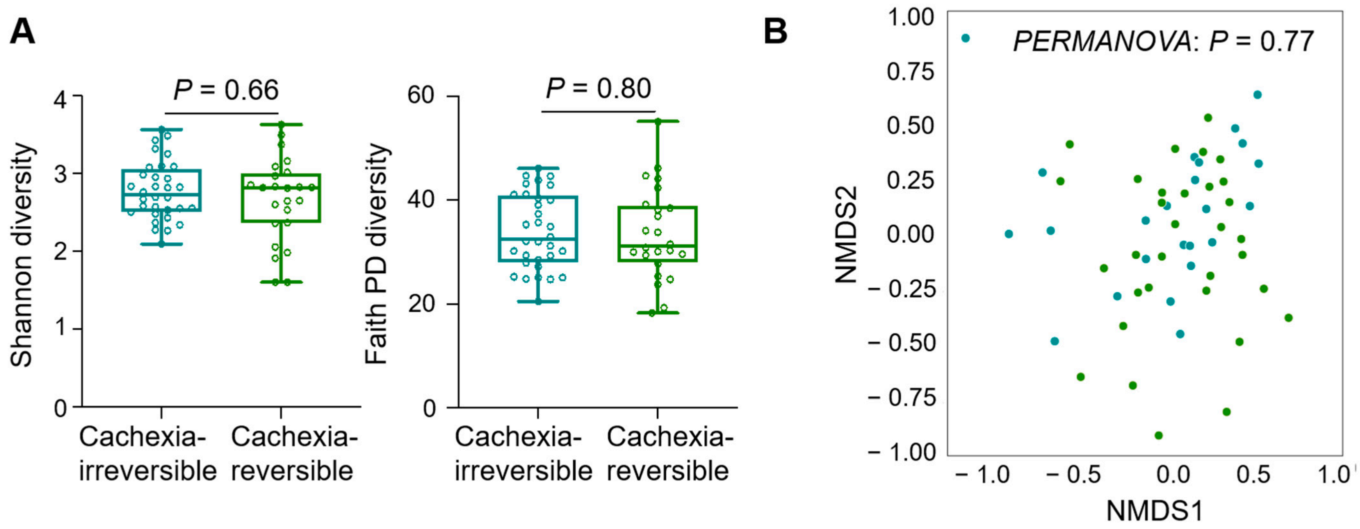

Figure S4. Differential analysis of GM stratified according to changes in the patients' longitudinal body weight during ICI therapy: (**A**) alpha diversity analysis with the Shannon and FaithPD indices, (**B**) ordination method-based nonmetric multidimensional scaling plots with Bray–Curtis distance.
